# A Novel Hyperthermostable Recombinant Protein Nanocage

**DOI:** 10.52547/ibj.3839

**Published:** 2022-10-29

**Authors:** Yaghoub Ahmadyousefi, Massoud Saidijam, Bagher Amirheidari, Fatehmeh Rahbarizadeh, Meysam Soleimani

**Affiliations:** 1 Department of Medical Biotechnology, School of Advanced Medical Sciences and Technologies, Hamadan University of Medical Sciences, Hamadan, Iran;; 2 Research Center for Molecular Medicine, School of Medicine, Hamadan University of Medical Sciences, Hamadan, Iran;; 3 Department of Pharmaceutical Biotechnology, Faculty of Pharmacy, Kerman University of Medical Sciences, Kerman, Iran;; 4 Extremophile and Productive Microorganisms Research Center, Kerman University of Medical Sciences, Kerman, Iran;; 5 Department of Medical Biotechnology, Faculty of Medical Sciences, Tarbiat Modares University, Tehran, Iran;; 6 Department of Pharmaceutical Biotechnology, School of Pharmacy, Hamadan University of Medical Sciences, Hamadan, Iran

**Keywords:** Ferritins, Proteins, Protein stability, Thermotolerance

## Abstract

**Background:**

Ferritin has an important role in iron storage in the cells, and due to its nanocage structure and self-assembly properties, it has wide application prospects in nanobiotechnology.

**Methods:**

The maize (*Zea mays*) ferritin gene *ZmFer1* was cloned and expressed in *Escherichia coli* BL21 (DE3) for the first time. Change in macromolecular structure of ZmFer1 ferritin due to heat treatment was investigated using native PAGE electrophoresis, DLS, and TEM. Change in the secondary structures of the protein was evaluated using CD spectroscopy. Moreover, alteration in the conformation of the protein was evaluated using UV-absorption spectra and intrinsic fluorescence spectra. The T_m_ of ZmFer1 was obtained using DSC. Finally, the effect of heat on the function of ZmFer1 was assessed by iron loading ability.

**Results:**

The purified ZmFer1 protein showed a homopolymer nanocage structure. The results of native PAGE electrophoresis, DLS, and TEM techniques showed that ZmFer1 protein nanocage is stable to heat treatment up to 90 °C, and some of the protein nanocages retain their macromolecular structures even at 100 °C in liquid aqueous solution. Based on the DSC results, ZmFer1 protein nanocage had a T_m_ of 81.9 °C. After treatment at 100 °C, stable ZmFer1 protein nanocages were able to store iron atoms.

**Conclusion:**

Recombinant ZmFer1 ferritin with a T_m_ > 80°C is a hyperthermostable protein nanocage. The results of this study are beneficial for the development of protein nanocages that are stable under extreme temperature conditions, as well as application of ZmFer1 in nanobiotechnology, biomaterials, and biomedical fields.

## INTRODUCTION

Ferritin is an essential iron storage protein in almost all living organisms (animals, plants, and bacteria). This protein can store iron in a soluble, nontoxic, and bioavailable form^[^^1^^]^. Ferritin is composed of 24 subunits that forms a protein nanocage structure to encapsulate iron atoms^[^^2^^]^. Due to the specific structure, ferritin can be used as an encapsulation and transport agent for nutrients (e.g. iron, calcium, and zinc), drugs, vitamins, and other bioactive organic substances in appropriate conditions^[^^3^^,^^4^^]^. Loading elements in ferritin not only help their storage but also increase their absorption in the body^[^^3^^]^. 

Protein nanocages have utility in vaccine design, targeted drug delivery, and synthetic biology^[^^5^^]^. Thermostable proteins are useful as biotemplates because they can withstand a wide range of processing conditions in the production of biomaterials^[^^6^^]^. The structure and function of these proteins are affected by temperature changes^[^^7^^]^. Identifying the mechanism of alterations in the physicochemical and functional properties of proteins due to heat treatment is now of interest to researchers. Many studies have reported the effects of heat treatment on the structural and functional properties of bioactive proteins^[^^8^^-^^14^^]^. Protein nanocages are biomaterials that their structural integrity and stability are of great importance for their usefulness in biological and nanotechnological applications. Changes in physicochemical and structural properties of protein nanocages due to heat treatment have been investigated by some researchers^[^^15^^,^^16^^]^. 

Maize, after wheat and rice, is the most important grain globally^[^^17^^]^. Compared to other grains such as wheat, rice, and soybean, maze is cheaper^[^^18^^]^. Using this inexpensive source to extract ferritin for application in nanobiotechnology, biomedical, and biomaterials can economically be beneficial. In this study, the maize ZmFer1 ferritin nanocage was obtained by heterologous expression in *Escherichia coli* and subsequently purified by affinity chromatography. Alterations in the physicochemical and functional properties of the ZmFer1 protein nanocage were then evaluated by different techniques. The aim of this study was to evaluate the stability of ZmFer1protein nanocage at high temperatures.

## MATERIALS AND METHODS

Materials

Maize (*Zea mays* L. cv. Single Cross 704) seeds were obtained from the Kerman Agricultural and Natural Resources Research and Education Center (Jiroft, Kerman, Iran). Plasmid pET28a, *E. coli* DH10b, and *E. coli* BL21 (DE3) were maintained in the laboratory of Dr. Soleimani at Hamadan University of Medical Sciences, Hamedan, Iran. Luria-Bertani medium was acquired from Ibresco Life Science (Karaj, Iran) and kanamycin from Sigma-Aldrich (San Diego, California, USA). IPTG was purchased from DNAbiotech (Tehran, Iran). Plasmid isolation kit, and 1-kb DNA ladder were procured from Thermo Fisher Scientific Inc. (USA). Ni-NTA agarose resin was from DNAbiotech. RNX-Plus RNA extraction kit was obtained from SinaClon (Tehran, Iran) and the first-strand cDNA synthesis kit was procured from Thermo Fisher Scientific Inc. All other compounds were from Sigma-Aldrich.

Expression and purification of ZmFer1 ferritin nanocage

The nine-day-old maize plantlets were removed from the soil, and the roots were washed with distilled water. Next, the plantlets were kept in a Petri dish at room temperature, and the roots were overlaid with two sheets of Whatman filter paper to keep the roots moist. After one day of starvation, plantlets were cut from the crown, and the stems dipped into the treatment solution (500 µM of FeSO_4_ and 1 mM of trisodium citrate). Leaf samples were harvested 6 h after treatment, frozen in liquid nitrogen, and stored at -80 °C. Total RNA was isolated from 100 mg of the frozen leaf samples using RNX-Plus (SinaClon) according to the manufacture’s recommendations and quantified by NanoDrop^TM^ 1000 spectrophotometer (Thermo Fisher Scientific). The cDNA was obtained by reverse transcription using the first strand cDNA synthesis kit (Thermo Fisher Scientific Inc.) with random hexamer primers following the manufacturer’s instruction. The coding region of *ZmFer1* was determined according to a previous study (GenBank: X61391.1)^[^^19^^]^. The coding region was amplified from the cDNA with a forward primer (F) containing a *Nco*I restriction site and a reverse primer (R) containing a *Bam*HI restriction site. PCR primers included F: CATGCCATGGGTCATC ATCACCATCATCACGCCGCGGGCAAGGGGAAGGAG and R: GGCCGCGCGGATCCAACATTACA CACTTGTTTCCTTCATTCATCTGTCC. The PCR products were gel purified and cloned into pET28a+ vector. Recombinant plasmid pET28a-ZmFer1 was then transformed into *E. coli* BL21 (DE3) and cultivated in Luria-Bertani medium to induce the expression of the recombinant ZmFer1 protein using IPTG. After ultrasonic lysis of the bacterial cells in an ice bath, the cleared lysate was obtained and centrifuged at 16000 × g at 4 °C for 20 min. The sediment was discarded, and the supernatant was applied to the Ni-NTA column. ZmFer1 was further purified with the sodium phosphate buffer (50 mM of NaH_2_PO_4_ and 300 mM of NaCl, pH 8) as the elution buffer. The purified ferritin was further dialyzed against the Tris-HCl buffer (20 mM, pH 8). Finally, the obtained ferritin was stored at 4 °C for further analysis.

Thermal treatment

ZmFer1 ferritin was diluted to the desired concentrations with 20 mM of Tris-HCl (pH 8), and samples were heated under different temperatures (50, 60, 70, 80, 90, or 100 °C) using a Thermo Block for 10 min. The heated samples were stored at 4 °C until additional analysis.

Polyacrylamide gel electrophoresis

SDS-PAGE was performed using the following procedure. Samples (50 µl) were mixed with the 5× Laemmli sample buffer (12.5 µl) containing 0.0875 M of Tris-HCl (pH 6.8), 45% (V/V) glycerol, 5% SDS, 12.5% (V/V) β-mercaptoethanol, and 0.01% bromophenol blue. After boiling for 5 min, the samples were centrifuged at high speed for 3 min and loaded onto a 12% SDS-PAGE gel. The electrophoresis was performed under a constant voltage of 120 V at room temperature. The gel was then stained with a staining solution containing 0.1% CBB R-250, 50% (V/V) methanol, and 10% (V/V) citric acid and destained in a destaining solution containing 50% (V/V) methanol and 10% (V/V) citric acid without CBB. The native PAGE was conducted through the following process. First, samples (50 µl) were mixed with the 4× native sample buffer (16.7 µl) containing 0.0875 M of Tris-HCl (pH 8) and 40% (V/V) glycerol without SDS and β-mercaptoethanol. Subsequently, the samples were loaded onto a 6% native PAGE gel without boiling,, and the electrophoresis was performed under a constant voltage of 120 V at 4 °C for 3 h. The gel was then stained with a staining solution containing 0.1% CBB R-250, 50% (V/V) methanol, and 10% (V/V) citric acid and destained in a destaining solution containing 50% (V/V) methanol and 10% (V/V) citric acid without CBB.

Western blotting

The SDS gel was made to transfer the protein bands on a polyvinylidene difluoride membrane for a subsequent immunodetection. The membrane was activated first by wetting in 100% methanol for a few seconds and then equilibrating in transfer buffer (25 mM of Tris and 192 mM of glycine (pH 8.3)) supplemented with 20% methanol (V/V) and 0.1% SDS for 10 minutes. The transfer process was carried out at 37 mA at 4 °C temperature for 13 h. The membrane was blocked in 3% nonfat dry milk in PBS-Tween 20 (0.1%) while shaking at room temperature for 1.5 h. Thereafter, the membrane was washed three times with PBS-Tween 20 (0.1%), using 5 min before being incubated with 7 µL of the horseradish peroxidase-conjugated anti-His antibody (Thermo Fisher) in 10 mL of PBS-Tween 20 (0.1%) for 1.5 h. After the incubation, the membrane was again washed four times with PBS-Tween 20 (0.1%) for 5 min. The blot was developed chromogenically with DAB (Sigma-Aldrich). The membrane was incubated with 10 mL of the DAB chromogen solution (0.6 mg/mL) until red/brown signals were detectable. Afterwards, the solution was discarded, and the membrane was dried in the dark.

Dynamic light scattering

Particle size was analyzed by DLS technique on a ZetaSizer Nano ZS instrument (Malvern, UK) at 25 °C with a laser beam wavelength of 633 nm. The backscattering mode was used for the analysis with the scattering angle of 173° and duration of 60 s. A minimum of three measurement runs was conducted on samples. 

Transmission electron microscopy

TEM was performed by Zeiss-EM10C-100 kV (Germany) at Day Petronic Company (Tehran, Iran). A drop of protein solution was placed onto the formvar/carbon-coated copper grids size 300 mesh (Electron Microscopy Sciences, USA) for 5 min, and uranyl acetate (2%) was then used to stain the sample for 30 seconds.

Circular dichroism

The secondary structure of the protein was measured by CD Spectropolarimeter (CD, J-810, Jasco Corp., Tokyo, Japan) at Razi University (Kermanshah, Iran). Samples were diluted to 38.4 µg/mL (1.55 µM of subunit protein) with 20 mM of Tris-HCl (pH 8) and transferred to quartz cuvettes with the optical path length of 1 cm. The spectra of CD were obtained from 200 nm to 260 nm with a 0.5-nm step and 1-nm bandwidth. The scan rate was 100 nm/min, and the temperature was set to 25 °C. The proportion of α-helix secondary structure was estimated in accordance with the previously reported methods^[^^20^^-^^24^^]^.

UV‐visible spectroscopy

The protein samples (500 µg/mL) were prepared and transferred to quartz cuvettes with an optical path length of 1 cm. Tris-HCl (20 mM, pH 8) buffer was used as the blank. The UV-visible absorbance spectra were obtained from 240 to 400 nm by a UV‐visible spectrophotometer (Specord 210 plus, Analytik-Jena, Germany)^[^^25^^]^.

Fluorescence measurements

The internal fluorescence of protein samples (500 µg/mL) was measured with a fluorescence spectrophotometer instrument (F-6200-Jasco, Japan). The excitation wavelength was 290 nm, and the fluorescence spectra were recorded from 300 to 500 nm. The excitation and emission bandwidths were set as 5 nm^[^^26^^]^.

**Differential scanning calorimetry**

The thermal denaturation temperature of the ZmFer1 protein nanocage was measured by DSC (TA-Q600, USA) at Beam Gostar Taban Laboratories (BGTL, Tehran, Iran). ZmFer1 ferritin (1 mg/mL) in 20 mM of Tris-HCl (pH 8) was prepared. Then, 1.965 mg of the protein solution was placed in the sample pan. The blank control was double distilled water. The heating temperature was set from 30 °C to 100 °C, at the rate of 5 °C/min.

**Iron staining**

Iron loading was performed by adding approximately 2,000 iron atoms to each ZmFer1 nanocage in five increments (1.92 µL of 80 mM FeSO_4_ solution, pH 2.0) at 0.5-h intervals. Electrophoresis was carried out in 6% native PAGE gels at a constant current of 6 mA (milliamps) for 10 h (at 4 °C) with a running buffer of 25 mM of Tris-glycine (pH 8.3). The Prussian blue method was performed for iron staining of the gels^[^^27^^]^. Gels were stained with a mixture of 2% potassium ferrocyanide (K₄Fe(CN)₆) and 2% hydrochloric acid (12.178 M; 1:1, V/V) for 1 h. The gels were then destained with distilled water and restained with CBB R-250 to stain proteins.

Statistical analysis

Data were presented as mean ± SD of at least three independent experiments and analyzed using SPSS software. A one-way analysis of variance (ANOVA) test was used to evaluate the statistical differences of two groups. *p *< 0.05 was considered statistically significant.

## RESULTS

Expression and purification of ZmFer1 protein 

**Fig. 1:**
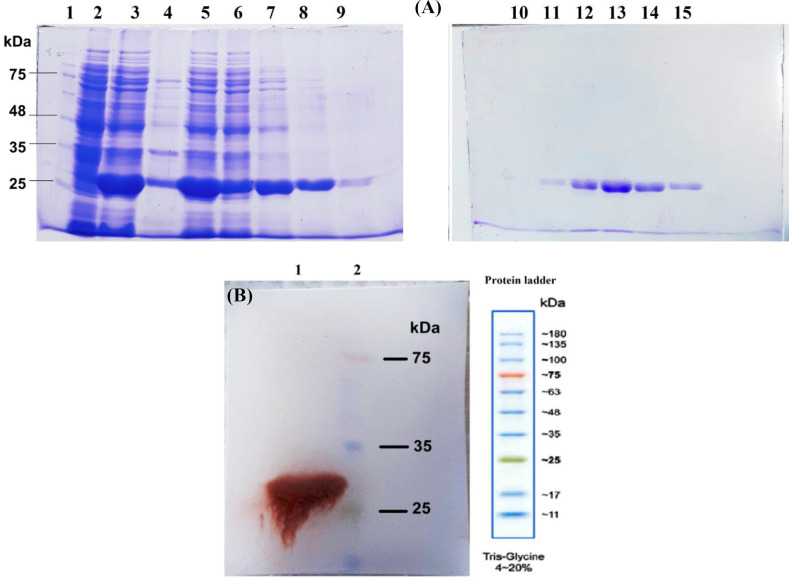
SDS-PAGE and Western blot analysis of the recombinant ZmFer1 protein nanocage after purification by affinity chromatography. (A) ZmFer1 was expressed in *Escherichia coli* BL21 (DE3) and purified by immobilized metal affinity chromatography using Ni-NTA resin. Lane 1, molecular weight protein ladder (SinaClon, Iran); lane 2, un-induced *E. coli* Bl21 (DE3) sample; lane 3, sonicated bacterial sample (cleared lysate); lane 4, cleared lysate pellet after centrifugation at 16,500 ×g and 4 °C (pellet); lane 5, cleared lysate supernatant after centrifugation at 20,000 ×g and 4 °C (supernatant); lane 6, the fraction containing proteins that failed to bind to the column (flow-through); lane 7, washing 1 (20 mM of imidazole); lane 8, washing 2 (40 mM of imidazole); lane 9, washing 3 (60 mM of imidazole); lanes 10-15, elutions 1-6, respectively. Conditions: 30 °C, 0.05 mM of IPTG, 20 h after induction. (B) Western blot profile for the purified ZmFer1 protein. Lane 1, purified ZmFer1 protein sample; lane 2, molecular weight protein ladder (SinaClon, Iran)

RNA was obtained from frozen leaves of the iron-treated maize plant shoots. The coding sequence of ferritin gene *ZmFer1* was amplified by PCR from the cDNA and cloned into pET28a plasmid. Cloning of*ZmFer1 *was verified by digesting the recombinant plasmid using restriction enzymes (Supplementary Fig. 1). Maize ZmFer1 ferritin was expressed in *E. coli* Bl21 (DE3) and purified by affinity chromatography using Ni-NTA column. Un-induced, cleared lysate, pellet, supernatant, flow-through, washing, and elution samples were analyzed by SDS-PAGE (Fig. 1A). According to SDS-PAGE results, ZmFer1 consisted of a subunit with a molecular weight of about 26 kDa (Fig. 1A). The expression of ZmFer1 protein was confirmed by Western blotting (Fig. 1B).

Effect of temperature on the macromolecular structure of ZmFer1 protein nanocage

**Fig. 2:**
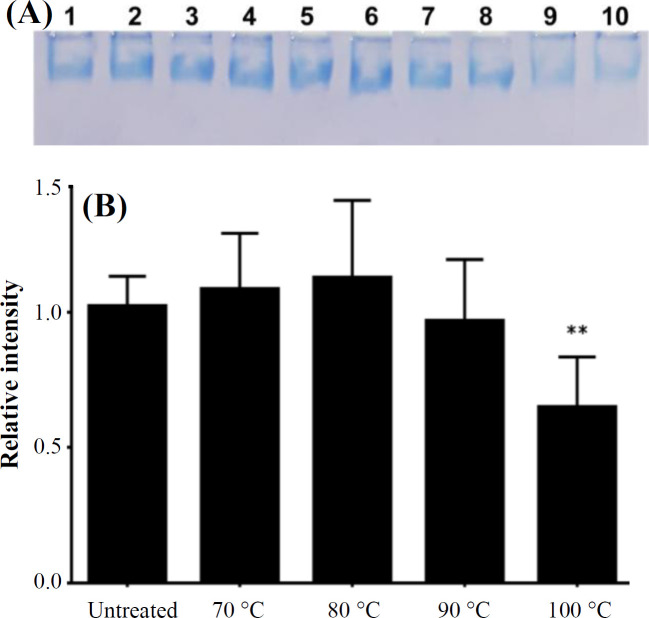
Native PAGE electrophoresis of ZmFer1 ferritin nanocage after heat treatment. (A) Representative image of native PAGE gel (6%) after electrophoresis and staining with CBB R250. Untreated ferritin (lanes 1 and 2); heat treatment of 70 °C (lanes 3 and 4), 80 °C (lanes 5 and 6), 90 °C (lanes 7 and 8), and 100 °C (lanes 9 and 10). (B) Band intensities were quantified using ImageJ software (National Institutes of Health, Maryland, USA) and reported relative to the band intensity of the untreated sample. Data are reported as mean ± SD (n = 10). ^**^p ≤ 0.01 compared with the untreated group based on One-way ANOVA results followed by Tukey HSD Post-hoc Test. No significant difference (*p *≥ 0.05) was observed between 70 °C, 80 °C or 90 °C and the untreated group. The difference between 70 °C, 80 °C or 90 °C, and the 100 °C-group was significant with *p values *of 0.0004, 0.0001 and 0.0140, respectively). Other comparisons (70 °C vs. 80 °C, 70 °C vs. 90 °C, and 80 °C vs.  90 °C) were not significant (*p *≥ 0.05) based on One-way ANOVA

**Fig. 3.**
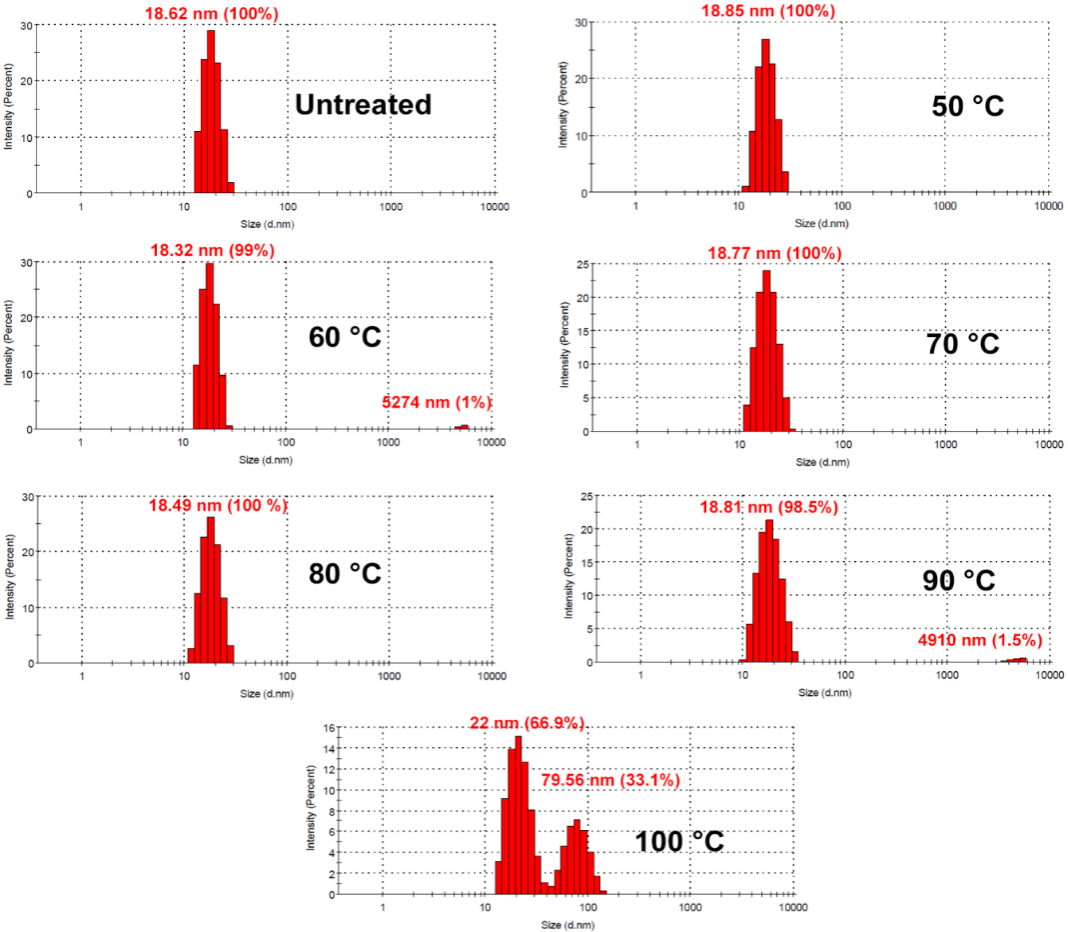
DLS analysis of ZmFer1 protein nanocage upon heat treatment. Samples were treated at different temperatures (room temperature for untreated sample, and 50-100 °C temperatures for other groups) and then centrifuged at 20,000 ×g at 4 °C for 100 min; Sediments were discarded, and supernatants were used for DLS analysis

**Fig. 4:**
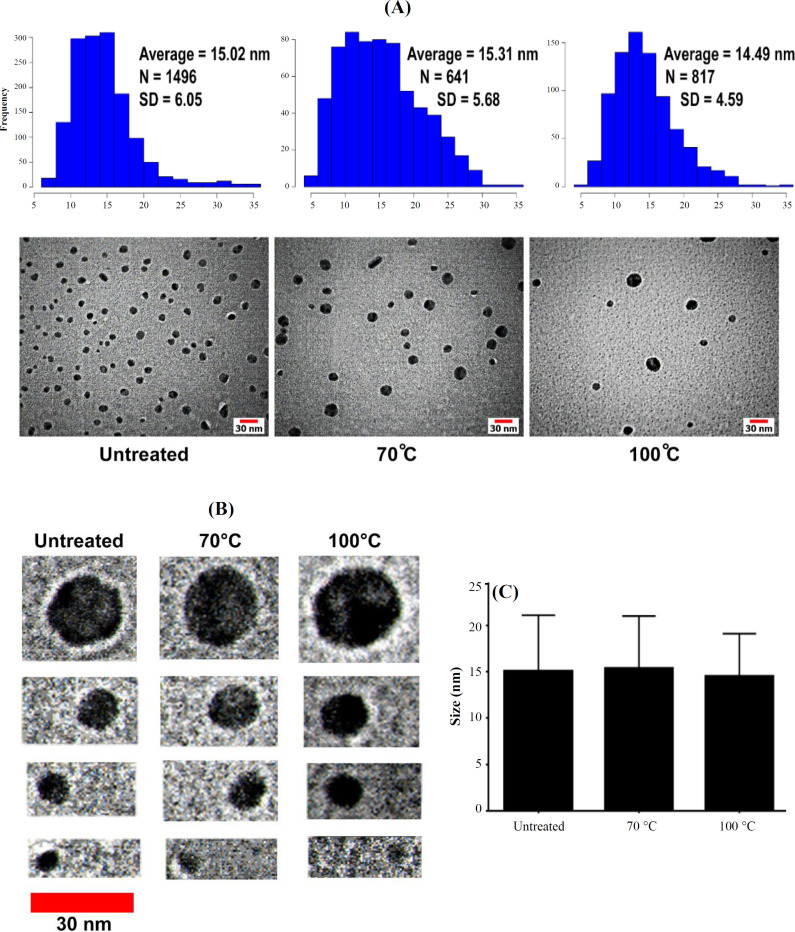
Morphology and size of ZmFer1 protein nanocages upon thermal treatments TEM images. (A) TEM images and histogram distribution plots of ZmFer1 nanocage under different thermal treatments (room temperature, 70 °C, and 100 °C), scale bars: 15 nm; (B) TEM images of different ZmFer1 hollow structures with different sizes under different thermal treatments (room temperature, 70 °C, and 100 °C), scale bar: 30 nm; (C) Size of ZmFer1 nanocages was quantified using ImageJ software (National Institutes of Health, Maryland, USA). Data are reported as mean ± SD (n = 1,496 for the untreated group, n = 641 for the 70 °C group, and n = 817 for the 100 °C group)

Native PAGE analysis was performed to evaluate the thermal stability of ZmFer1 nanocage. The intensity of the ZmFer1 protein band was constant up to 90 °C and decreased significantly at 100 °C, indicating partial denaturation under this condition (Fig. 2). Based on the results of native PAGE electrophoresis, ZmFer1 protein nanocages maintained their macromolecular state up to 90 °C, and some of the protein nanocages retained their macromolecular structure even at 100 °C (Fig. 2). The effect of thermal treatment on the assemble properties of ZmFer1 protein nanocage was investigated by DLS (Fig. 3). Protein samples were centrifuged at 20,000 × g at 4 °C for 100 min before performing the DLS technique. After centrifugation, the DLS signal of the protein nanocage was observed in all treatment groups, i.e. untreated group and the groups with heat treatment at 50-100 °C (Fig. 3 and Table 1). At 50-90 °C, the only recorded signal was related to the nanocage with a hydrodynamic size of 15-22 nm. However, at 100 °C, another peak for soluble aggregated particles was observed with an average size of 79.56 nm (Fig. 3 and Table 1). TEM was used to evaluate the quaternary structure as well as the spherical shell structure of ZmFer1 protein nanocage after heat treatment (Fig. 4). The nanocage structure of ZmFer1 ferritin was stable under different thermal treatments (room temperature, 70 °C and 100 °C). Untreated ZmFer1 proteins had a spherical nanocage structure with an average outer diameter of about 15.02 nm. The average outer diameter of spherical nanocage structures at 70 °C- and 100 °C-treated protein samples were about 15.31 nm and 14.49 nm, respectively (Fig. 4A). 

There were different types of spherical hollow structures with different sizes in the TEM images of ZmFer1 protein, observed in all heat treatment groups (untreated, 70 °C-treated and 100 °C-treated samples; Fig. 4B). The size of nanocages was not significantly different among the untreated group, 70 °C, and 100 °C-treated groups (Fig. 4C).

Effect of temperature on the secondary structure of ZmFer1 

**Fig. 5:**
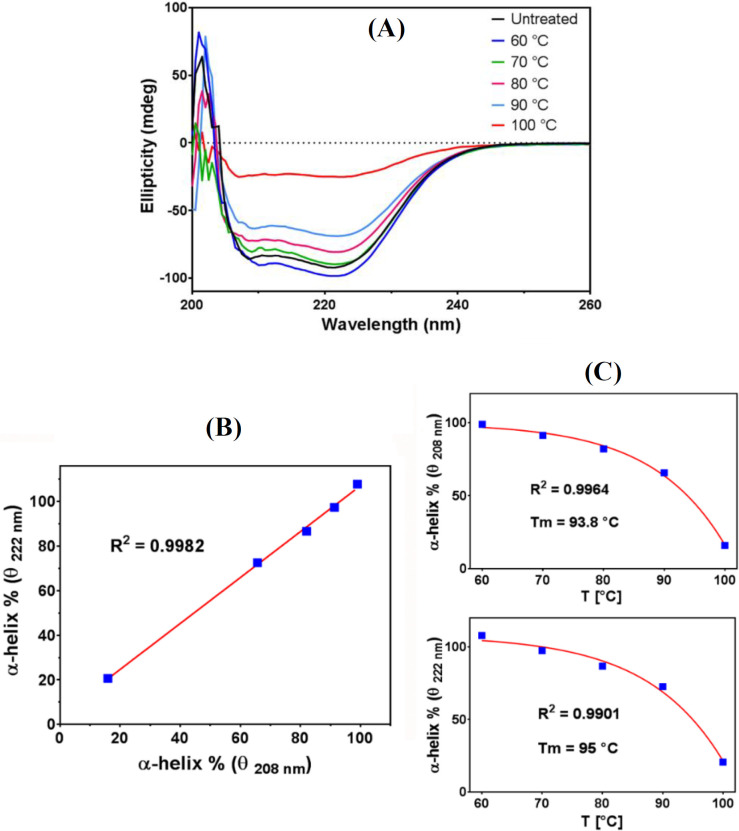
CD analysis of ZmFer1 protein nanocage after heat treatments.** (**A) CD spectra of ZmFer1 protein nanocage after heat treatment at different temperatures (60-100 °C) for 10 min, with untreated protein as a control; (B) correlation between the percentage of α-helix secondary structure calculated based on CD spectra at 208 and 222 nm; θ, observed ellipticity (mdeg); (C) The calculated average fraction of folded α-helix structures in ZmFer1 ferritin based on θ_ 208 nm_ and θ _222 nm_ at different temperatures (60-100 °C). Data were fitted with a Boltzmann sigmoidal model using GraphPad Prism (GraphPad Software Inc, California, USA)

The conformational changes of ZmFer1 protein nanocage due to heat treatment at different temperatures were determined by CD spectroscopy (Fig. 5A). There were two clear negative peaks in the far-UV region of untreated ZmFer1 CD spectra, mainly at 208 and 222 nm, indicating that the secondary structure of ZmFer1 mainly comprises the α-helix. Based on the CD spectroscopy, the ellipticity at 222 nm and 208 nm decreased with increasing temperature. The contents of the α‐helix secondary structures in ZmFer1 protein nanocage were calculated using the following equations^[^^20^^-^^24^^]^:

$\mathrm{MRE}=\frac{\theta }{c\times n\times l\times 10}$

α-Helix%=-MRE208-400029 000×100

α-Helix%=-MRE222-234030 300×100

Where, MRE is mean residue ellipticity (deg cm^2^ dmol^-1^), θ is the observed ellipticity (mdeg), c is the molar concentration of protein (herein 1.55 × 10^-6^ mol/L), n is the number of amino acid residues of protein (herein 216), l is the path length of the quartz cell (herein 1 cm), and MRE_208_ and MRE_222_ are the calculated mean residue ellipticity values at 208 and 222 nm, respectively. The calculated content of α-helix structures based on the CD spectra at 208 nm was correlated to the calculated value at 222 nm (*p* < 0.0001; Fig. 5B). To estimate the fraction of folded α-helix structures in ZmFer1 protein during treatment with different temperatures, the calculated content of α-helix secondary structures based on the CD spectra at 208 and 222 nm were investigated (Fig. 5C). Data were fitted with a Boltzmann sigmoidal model using GraphPad Prism (GraphPad Software Inc, California, USA)^[28]^. The results showed that heat treatment reduced the fraction of folded α-helix structures in ZmFer1 protein (Fig. 5C). Based on the fitted Boltzmann sigmoidal model (R^2^ = 0.9964 and R^2^ = 0.9901 for calculations based on θ_ 208 nm_ and θ _222 nm_, respectively), a T_m_ of 93.8-95 °C was calculated for the folded α-helix secondary structures in ZmFer1 ferritin nanocage.

UV absorbance and fluorescence change of ZmFer1 ferritin

**Fig. 6:**
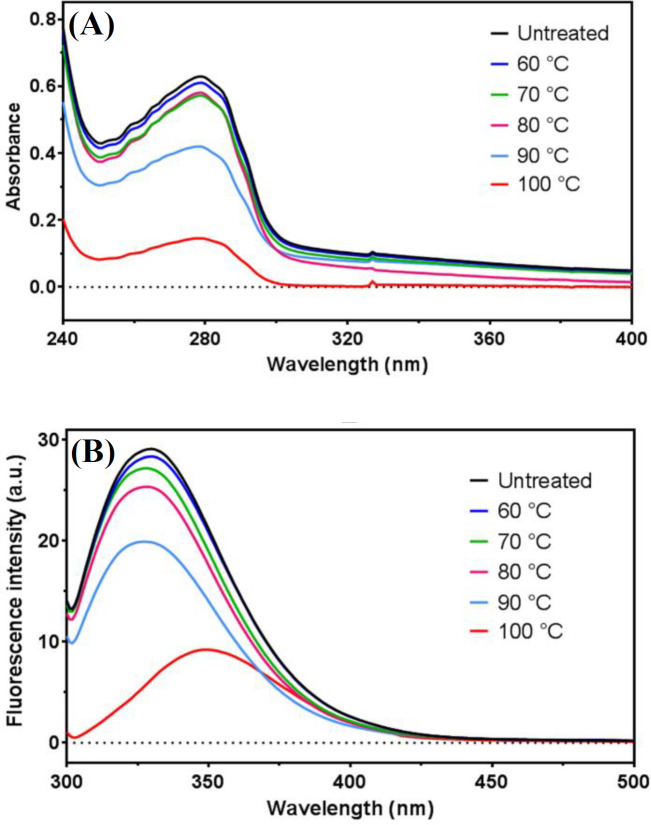
Changes in UV spectra and fluorescence spectra of ZmFer1 protein nanocage after heat treatments. (A) UV spectra of ZmFer1 ferritin upon heat treatment at different temperatures (60-100 °C) for 10 min, with untreated protein as a control; (B) fluorescence spectra of ZmFer1 ferritin (λ _Excitation_ = 290 nm) after heat treatment at different temperatures (60-100 °C) for 10 min, with untreated protein as a control

As shown in Figure 6A, with increasing temperature, the absorption at 280 nm gradually decreased. As the temperature rises to 100 °C, the unique peak at 280 nm is still visible (Fig. 6A). At 90 °C and 100 °C, the UV absorption peak significantly reduced. The fluorescence spectra of ZmFer1 protein nanocage at 290 nm is shown in Figure 6B. Untreated and 60-90 °C-treated ZmFer1 proteins had a fluorescence peak with λ_max_ of 330 nm. At 100 °C, the fluorescence emission peak decreased with a red shift (λ_max_ = 350 nm; Fig. 6B). 

**T**_m_ **of ZmFer1 protein nanocage**

**Fig. 7:**
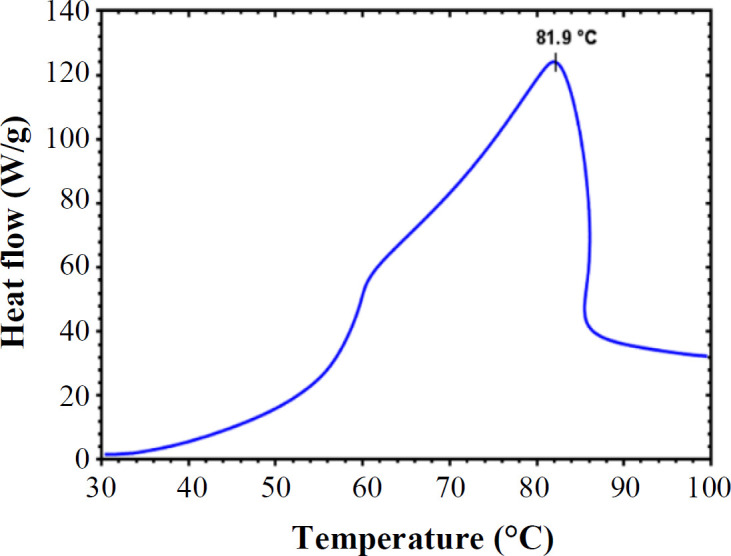
Determination of thermal stability of ZmFer1 protein nanocage using DSC. The heating temperature was set from  30 °C to 100 °C, at the rate of 5 °C/min

DSC is an analytical technique that can provide information about the thermal denaturation temperature of a protein. Based on DSC technique, the T_m_ of ZmFer1 protein nanocage was 81.9 °C in liquid aqueous solution (Fig. 7).

**Effect of temperature on iron loading ability of ZmFer1 ferritin nanocage**

**Fig. 8:**
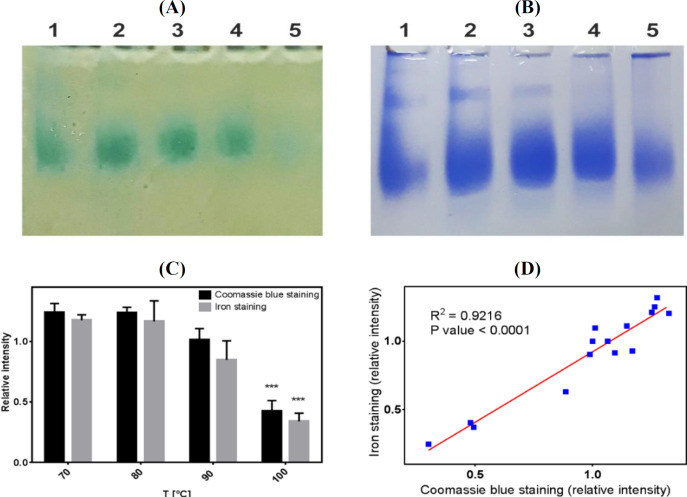
Iron loading assay of ZmFer1 protein nanocage after heat treatments. (A) Iron staining by the Prussian blue method after native PAGE electrophoresis. (B) protein staining CBB R-250. Untreated ferritin (lane 1); heat treatments at 70 °C (lane 2), 80 °C (lane 3), 90 °C (lane 4), and 100 °C (lane 5); (C) relative band intensities were quantified using ImageJ software (National Institutes of Health, Maryland, USA). Data are reported as mean ± SD (n = 3); (D) positive correlation between iron staining and protein staining. Correlation was determined by linear regression using GraphPad Prism (GraphPad Software Inc, California, USA)

Recombinant ZmFer1 ferritin nanocage was tested for iron loading ability (Fig. 8). Iron loading caused protein aggregation in all samples. Protein aggregation increased with the elevation in iron concentrations. We performed iron loading experiment using 2,000 iron atoms per ZmFer1 nanocage in five steps with an interval of 0.5-h. Samples were centrifugated to remove protein aggregates. Based on the iron staining and protein staining techniques after native PAGE, ZmFer1 had the ability for iron loading even after heat treatment at different temperatures (70-100 °C; Fig. 8A-8C). Based on the results, the content of the loaded iron was positively correlated with that of ZmFer1 protein (Fig. 8D). 

## DISCUSSION

Ferritin is an iron storage protein in cells that forms a protein nanocage structure. Plant ferritin represents a novel class of iron and nutrient supplement factors, which is a promising protein nanocage for different applications in nanobiotechnology, biomaterials, and biomedical fields. In this study, the maize ZmFer1 ferritin nanocage was cloned and expressed in *E. coli* BL21 (DE3) for the first time, and its thermal stability was evaluated. 

The protein subunit of ferritin can be assembled into a protein cage with biological activity, but this property will be lost upon aggregation^[^^29^^]^. In our study, the macromolecular structure of ZmFer1 ferritin nanocage after heat treatment was evaluated using native PAGE, DLS, and TEM techniques. Native PAGE showed that ZmFer1 protein basically remained stable at 90 °C and the temperatures lower than it. However ZmFer1 protein partially denatured. with an increase in temperature up to 100 °C, Notably, some ZmFer1 nanocages could keep their unique macromolecular state at 100 °C. 

Based on DLS results, the peak of protein nanocage with a hydrodynamic size of 15-22 nm was observed in all temperature groups (50-100 °C temperatures). TEM results showed that ZmFer1 protein maintains its nanocage structure at different temperatures, even at 100 °C. The size and morphology of nanocages were not significantly different among the untreated, 70 °C-, and 100 °C-treated samples. According to TEM experiments, oyster ferritin was stable only up to 80 °C when was heated for 10 min^[^^15^^]^. At 90 °C, the spherical shell structure of oyster ferritin reduced, and at 100 °C, the spherical shell structure was not observable in TEM images^[^^15^^]^. Tang et al.^[^^16^^]^ heated pea seed ferritin at different temperatures (60-100 °C) for 10 min. Although the mentioned ferritin was stable up to 80 °C, native PAGE showed the instability of pea seed ferritin at 90 and 100 °C^[^^16^^]^. These results were confirmed by TEM experiments. TEM results showed that pea seed ferritins were put together at 90 °C, and shell-like structures were hardly observed at 100 °C^[^^16^^]^.

**Table 1:** Results of DLS analysis of ZmFer1 protein nanocage after heat treatments

**Treatment** **(°C)**	**DLS analysis**				
	**Nanocage**			**Aggregates**	
	**Size (nm) ± SD**	**Intensity (%)**		**Size (nm) ± SD**	**Intensity (%)**
Untreated	18.62 ± 3.42	100		-	0
50	18.85 ± 3.75	100		-	0
60	18.32 ± 3.20	99		5274 ± 422.7	1
70	18.77 ± 4.19	100		-	0
80	18.49 ± 3.79	100		-	0
90	18.81 ± 4.71	98.5		4910 ± 661.2	1.5
100	22.00 ± 5.74	66.9		79.56 ± 20.04	33.1

Samples were treated at different temperatures (room temperature for untreated sample, and 50-100 °C temperatures for other groups) and centrifuged at 20,000 ×g for 100 min at 4 °C. Sediments were discarded, and supernatants were used for DLS analysis. DLS was repeated three times with similar results.

The results of native PAGE, DLS, and TEM demonstrated that ZmFer1 ferritin nanocage is hyperstable to heat treatment and maintains its macromolecular structure at different temperatures, even in part at 100 °C. In this study, we could easily observe spherical shell-like structures of ZmFer1 in TEM images after treatment at 100 °C. While simple assemblies can be analyzed with conventional electron microscopy images^[^^30^^]^, it is too difficult to analyze the assembly of complex proteins such as ferritinwith 24 subunits using electron microscopy images. Different cross sections of these complex protein assemblies in TEM images leads to different patterns that makes it difficult to analyze the macromolecular structure of these complexes using TEM images. ZmFer1 is a homopolymer protein nanocage. In nature, ferritins are also heteropolymer protein nanocages with two different types of subunits. Based on TEM images, it appears that the recombinant ZmFer1 subunit protein forms different types of homopolymer protein nanocages with different sizes. Larger hollow structures may be assemblies of two or more protein nanocages. Some of spherical hollow structures (probably the average ones) may be composed of 24 subunits as previously characterized for ferritin proteins in some organisms. Nanocages with smaller sizes are likely to be composed of fewer subunits. However, the details of the self-assembly properties of ZmFer1 protein nanocages require X-ray crystallography for confirmation and detailed examination. 

CD spectroscopy is a method to determine the secondary structures of proteins. Negative peaks at 208 and 222 nm of CD spectra are the results of n-π* charge transfer in the peptide bond of α‐helix and are used to calculate the content of α-helix structures in proteins^[^^31^^]^. The reduction of α-helix secondary structures leads to a conformational change in the subunit protein of ferritin that directly affects the nanocage structure of ferritin and reduces the stability of ferritin, leading to the irreversible unfolding of the protein. In our study, heat treatment reduced the fraction of folded α-helix structures in ZmFer1 protein with a T_m_ of 93.8-95 °C based on the calculations by θ _208_ nm and θ _222_ nm. These results were consistent with the previous experiments on oyster ferritin^[^^15^^]^ and pea seed ferritin^[^^16^^]^. In these studies, the content of α-helix structures in ferritin was reduced by heat treatment. In the case of oyster ferritin, at 100 °C, peaks at 208 and 222 nm were hardly observed, indicating that the structure of oyster ferritin was almost completely destroyed at this temperature^[^^15^^]^. In contrast, our results showed that the negative peaks at 208 and 222 nm of the CD spectra of ZmFer1 were visible even at 100 °C, suggesting that some ZmFer1 protein nanocages were still stable under these conditions. Moreover, it is known that conformational changes in proteins can alter UV absorption spectra^[^^32^^]^. The alteration in absorption at 280 nm in proteins depends on the change in the number of aromatic amino acids exposed to the solvent, especially tryptophan and tyrosine^[^^33^^]^. In our study, the UV absorption peak of ZmFer1 reduced significantly at 90 °C and 100 °C. The decrease in absorbance at 280 nm is due to the conformational changes in the ZmFer1 ferritin. These results showed that at 90 °C, a change in conformation of ZmFer1 protein occurred, leading to a change in the environment of aromatic amino acids. These results were in line with a previous study conducted on oyster ferritin^[^^15^^]^. As temperature increased, the UV absorbance peak of oyster ferritin decreased, and similar to our result, this phenomenon was more obvious at 90 and 100 °C^[^^15^^]^. However, in our study, the intensity of the UV absorbance signal in protein samples treated at 90 and 100 °C compared to untreated samples were higher than that of oyster ferritin, indicating the higher thermal stability of ZmFer1 protein nanocage than oyster ferritin. 

The residues of tryptophan, tyrosine, and phenylalanine in proteins have a fluorescence emission when excited at the UV light from 280 to 305 nm^[^^34^^]^.

The fluorescent peaks of phenylalanine, tryptophan, and tyrosine are located at 282 nm, 348 nm and 303 nm, respectively^[^^34^^]^. Various fluorescence spectra were generated due to different side chains of the exposed amino acids. In the present study, the ZmFer1 protein nanocage samples were exited at 290 nm after exposure to different temperatures. The λ_max_ of the fluorescence spectra for untreated and the 60-90 °C groups were unchanged (λ_max_ = 330 nm). As temperature raised, the fluorescence emission peak of protein quenched, and this fluorescence quenching was more obvious at 90 and 100 °C. However, at 100 °C, the fluorescence spectrum of ZmFer1 ferritin was still visible with a red shift in fluorescence (λ_max_ = 350 nm). The fluorescence quenching phenomenon and the red shifted fluorescence at 100 °C may be due to a change in the structure of ZmFer1 ferritin, which is consistent with the results of CD spectroscopy in our study. An investigation on oyster ferritin showed that once temperature increases, the fluorescence emission is quenched with a red shift. Similarly, this phenomenon was even more obvious at 90 and 100 °C ^[^^15^^]^. However, in the present study, the intensity of the fluorescence signal in the protein samples treated at 90 and 100 °C compared to the untreated samples were higher than that of oyster ferritin, indicating the higher thermal stability of ZmFer1 protein nanocage than oyster ferritin. DSC measures the T_m_ and the energy required to disrupt the interactions stabilizing the structure of a protein^[^^35^^]^. In our study, based on the DSC results, the T_m_ of ZmFer1 protein nanocage was 81.9 °C. By contrast, the T_m_ of recombinant oyster ferritin was 76 °C based on DSC, which is lower than the ZmFer1 ferritin nanocage^[^^15^^]^. In addition, the T_m_ of ZmFer1 protein nanocage was higher than that of human H ferritin homopolymerwith a T_m_ of 77 °C using DSC^[^^36^^]^. 

The main function of ferritin is its ability to store iron atoms. In order to evaluate the iron loading ability of ZmFer1 after heat treatment, we used the iron loading assay. In the current study, we demonstrated that after heat treatment at different temperatures (50-100 °C) and subsequent centrifugation to remove protein aggregates, stable protein nanocages (heat-treated protein nanocages) retained their ability to load iron atoms. We also exhibited that this iron loading is correlated with the protein content at all temperature groups. This finding implies that different temperatures have no effect on iron loading capacity. We found that iron loading can induce the aggregation of ZmFer1 protein. Indeed, reduction in iron loading, as depicted in Figures 8A and 8C, is related to partial protein instability at 100 °C, iron-induced protein aggregation and not the iron loading capacity. Based on our results, ZmFer1 has the ability for iron loading and storage even after heat treatment at different temperatures (70-100 °C). Our results also showed that content of the loaded iron is positively correlated with the content of ZmFer1 protein, indicating that ZmFer1 ferritin nanocage maintained its main function to store iron atoms after exposure to different temperatures (70-100 °C).

Thermostable proteins are resistant to changes in protein structure due to the applied heat. In this study, we cloned and expressed a subunit of maize ferritin (ZmFer1), which forms a homopolymer protein nanocage. Native PAGE, DLS, TEM, CD, absorbance/fluorescence spectroscopy, and iron loading assay disclosed that the ZmFer1 protein nanocage could be stable up to 90 °C, and even at 100 °C, some protein nanocages were still stable. We could not examine the stability of ZmFer1 protein at temperatures above 100 °C. In a liquid aqueous solution, the temperature cannot be increased to more than 100 °C without increasing the pressure. We were unable to lyophilize ZmFer1 protein to examine the stability of the solid lyophilized ZmFer1 protein at the temperatures greater than 100 °C. 

DSC showed a T_m_ of 81.9 °C for the ZmFer1 protein nanocage in liquid aqueous solution. In this context, hyperthermostable proteins in the literature had a T_m_ > 80°C^[37-39]^. Moderate thermophiles are defined as microorganisms that optimally grow at temperatures above 45 °C and below 80 °C, and hyperthermophiles are defined as the microorganisms that optimally grow at temperatures above 80 °C^[^^40^^,^^41^^]^. According to this classification, we propose that the ZmFer1 protein nanocage, with a T_m_ of 81.9 °C, which is stable at temperatures up to 90 °C, and partial stable at 100 °C, is a hyperthermostable protein nanocage. 

This study evaluated the thermal stability of maize ZmFer1 ferritin nanocage in liquid aqueous solution. Based on the results of native PAGE electrophoresis, DLS, and TEM, ZmFer1 protein nanocage is stable to heat treatment up to 90 °C. At 100 °C, the protein nanocage possessed a relative thermostability, since some protein nanocages retained their macromolecular structure after 10 min at 100 °C. The results of CD spectroscopy showed that with increasing temperature, the fraction of folded α-helix secondary structures in the protein decreased with a T_m_ of 93.8-95 °C. UV absorbance and fluorescence spectroscopy indicated that at temperatures of 90 °C and 100 °C, a significant change in the absorbance and emission spectra occurs, which may be due to an alteration in the conformation of ZmFer1 protein that leads to a change in the environment of aromatic amino acids. ZmFer1 protein nanocage had a T_m_ of 81.9 °C using DSC. ZmFer1 protein nanocage was able to store iron after exposure to high temperatures up to 100 °C. We propose that the ZmFer1 protein nanocage is a hyperthermostable protein nanocage. The high stability of this protein nanocage to heat has made it a suitable nanocarrier that can reduce the costs of storage and transportation of the final product. In addition, this protein nanocage can be used in biomaterial processes where heat treatment is required.

## DECLARATIONS

### Acknowledgments

This research was extracted from a Ph.D. thesis (Yaghoub Ahmadyousefi) at the Medical Biotechnology Department, Hamadan University of Medical Sciences, Hamadan, Iran. Authors thank the Research Deputy of Hamadan University of Medical Sciences for supporting the research (grant no. 9804112683). We also are extremely grateful to all those who helped us in conducting this study, including the staff of Hamadan University of Medical Sciences.

### Ethical statement

This study was approved by the Research Ethics Committee of Hamadan University of Medical Sciences, Hamadan, Iran (ethical code: IR.UMSHA.REC.1398.186). 

### Data availability

The analyzed data sets generated during the study are available from the corresponding author on reasonable request.

### Author contributions

YA: Conceptualization, methodology, data curation, investigation, formal analysis, visualization, validation, and writing original draft; MS: resources, supervision, writing, review, and editing; BA: formal analysis, writing, review, and editing; FR: formal analysis, writing, review, and editing; MS: resources, validation, writing, review, editing, supervision, project administration, and funding acquisition.

### Conflict of interest

None declared.

### Funding/support

This study was financially supported by the Vice-chancellor for Research and Technology, Hamadan University of Medical Sciences, Hamadan, Iran (grant no. 9804112683). 

## Supplementary Materials


