# Supplementary material for: A Novel Hyperthermostable Recombinant Protein Nanocage
Source: Iran Biomed J. 2022 Oct 29;26(6):426–39. doi: 10.52547/ibj.3839 (PMC9841219; doi:10.52547/ibj.3839)
Supplement: Supplementary file 1 [file ibj-26-426-s1.pdf]

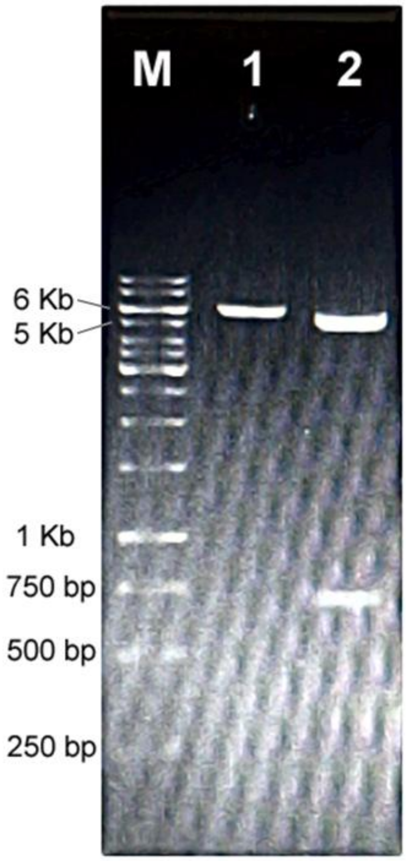

Supplementary Fig. 1. Validation of cloning by digestion of the recombinant plasmid using restriction enzymes. Lane M, 1-Kb DNA ladder (Thermo Fisher Scientific); lane 1, single digestion of the recombinant plasmid pET28a-ZmFer1 by *Nco*I; lane 2: double digestion of the plasmid pET28a-ZmFer1 using *Bam*HI and *Nco*I. Theorical length of digested fragment: 698 bp.
